# Success in Life

**Published:** 1882-08

**Authors:** 


### SUCCESS IN LIFE.

If to obtain wealth is success, we see men around us who have accumulated fortunes, who have no remarkable talent, no special high moral character; in fact, in general intelligence, in elevation of sentiment, in breadth of view, they are pitifully deficient; while men, immeasurably their superiors in every great and good quality, have never made and saved a dollar.

Then again, every now and then, we meet with a man who seems to have prospered in everything he ever attempted, while his next door neighbor, apparently in everything his equal, if not his superior, fails in every undertaking; every effort to rise is sure to result in a more hopeless fall.

Able and worthy men ought not to feel discouraged nor cast down, nor to whelm themselves with self-mistrust or self-reproaches; for the very foolhardiness of some men, and the stupidity of others, in not seeing palpable obstacles and dangers, is the father of their successes, while every succeeding one is the result of that *morale*, as the French term it, which attaches itself to great accomplishments.

In very many cases, the accumulation of fortunes is the merest chance; the result of a fire, or famine, or flood, or pestilence, or sword, or from inheritance; in such, there is no sort of credit due to pecuniary success.

In other cases, men make money by virtue of that utter abnegation of all moral principle which belongs to the most depraved mind; temptations to which debasements frequently present themselves to the noble-hearted, but are spurned the moment they are proffered, and are rejected without an effort, for it is far sweeter to them to live in destitution, than to dress in fine linen and fare sumptuously every day, at the cost of self-degradation and of prostituted honor.

There are a few men, however, who grow rapidly rich by the force of a perspicacity, a singleness of purpose, and an energy of will, which would have made them distinguished in any department of human life, in any pursuit to which they may have been directed; upon such men we ought not to look with envy, but with respect, and, while we should admire them the more, we ought not to think of ourselves the less, for all the great pecuniary difference, as long as we have been fast in our integrity in every strait, and in every temptation.

But suppose we have failed a dozen times, who knows but that it may be with us as it has been with multitudes before us, that past adverses are the foundations, constitute the elements, of future success, the very schoolings to great accomplishments.

Let every man, then, be diligent, and abide his time in patience, remembering that the race is not commonly, in practical life, to the swift, nor the battle to the strong; and that ultimate and permanent success, is the pretty sure reward of him, who has patience, diligence, and a great heart.

